# Intralobar pulmonary sequestration presenting as multiple nodular pulmonary lesions and focal emphysema

**DOI:** 10.1002/rcr2.1276

**Published:** 2024-02-01

**Authors:** Tadatsugu Yoshinaga, Makiko Yomota, Kazutoshi Toriyama, Hirokazu Iso, Kie Mirokuji, Shoko Kawai, Kosuke Narita, Mikito Suzuki, Hirotoshi Horio, Yukio Hosomi

**Affiliations:** ^1^ Department of Respiratory Medicine Tokyo Metropolitan Cancer and Infectious Diseases Center Komagome Hospital Tokyo Japan; ^2^ Department of Thoracic Surgery Tokyo Metropolitan Cancer and Infectious Diseases Center Komagome Hospital Tokyo Japan

**Keywords:** intralobar pulmonary sequestration, radiology and other imaging, rare lung diseases

## Abstract

The features of intralobar pulmonary sequestration vary on computed tomography (CT). Many cases demonstrate a mass or cystic lesion within a lower lobe. We report herein a case of a 55‐year‐old, female patient presenting with right back pain. Contrast enhanced (CE) CT revealed multiple, nodular, pulmonary lesions suggesting recurrent infections with surrounding focal emphysema. Three‐dimensional (3D) reconstruction demonstrated a sequestrated lung segment with a systemic, arterial blood supply. Based on these findings, intralobar pulmonary sequestration was diagnosed. Intralobar pulmonary sequestration can present as multiple, nodular, pulmonary lesions with focal emphysema rather than as a mass or cyst. CE‐CT with 3D reconstruction is useful for diagnosing this condition. Patients with recurrent pulmonary infections have a high index of suspicion of intralobar pulmonary sequestration.

## Graphical abstract

Intralobar pulmonary sequestration can present as multiple, nodular, pulmonary lesions with focal emphysema. CE‐CT with 3D reconstruction is useful for diagnosing this condition

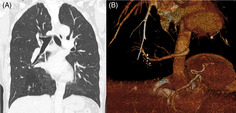

## INTRODUCTION

Pulmonary sequestration (PS) is a rare, congenital, lung anomaly consisting of non‐functioning pulmonary tissue lacking communication with the tracheobronchial tree and deriving its blood supply from the aorta or its branches and various forms of venous return.^1^ It is customarily considered a form of intralobar sequestration (ILS) or extralobar sequestration (ELS). The former occurs within the visceral pleura and is surrounded by normal lung tissue whereas the latter has its own pleura completely separating it from normal lung tissue. ILS and ELS accounts for 75%–90% and 10%–20% of PS cases, respectively.^2^

In ILS, recurrent pulmonary infections within a lower lobe are the most common presentation; tracheobronchial drainage fails to function properly.^3^

The computed tomography (CT) findings of ILS vary due to inflammation and infection.^4^ ILS presents rarely multiple, nodular, pulmonary lesions with focal emphysema surrounding the lesions. Identification of a systemic, arterial blood supply is key to diagnosing this condition. Normally, angiography is the chief method used for this purpose, but recently, contrast enhanced (CE)‐CT with multiplanar and three‐dimensional (3D) reconstruction has begun to be used as a more accurate, non‐invasive method of achieving this aim.^5^

We report herein a case of ILS presenting multiple, nodular, pulmonary lesions with focal emphysema. CE‐CT with multiplanar and 3D reconstruction proved to be useful in diagnosing the present case.

## CASE REPORT

**FIGURE 1:**
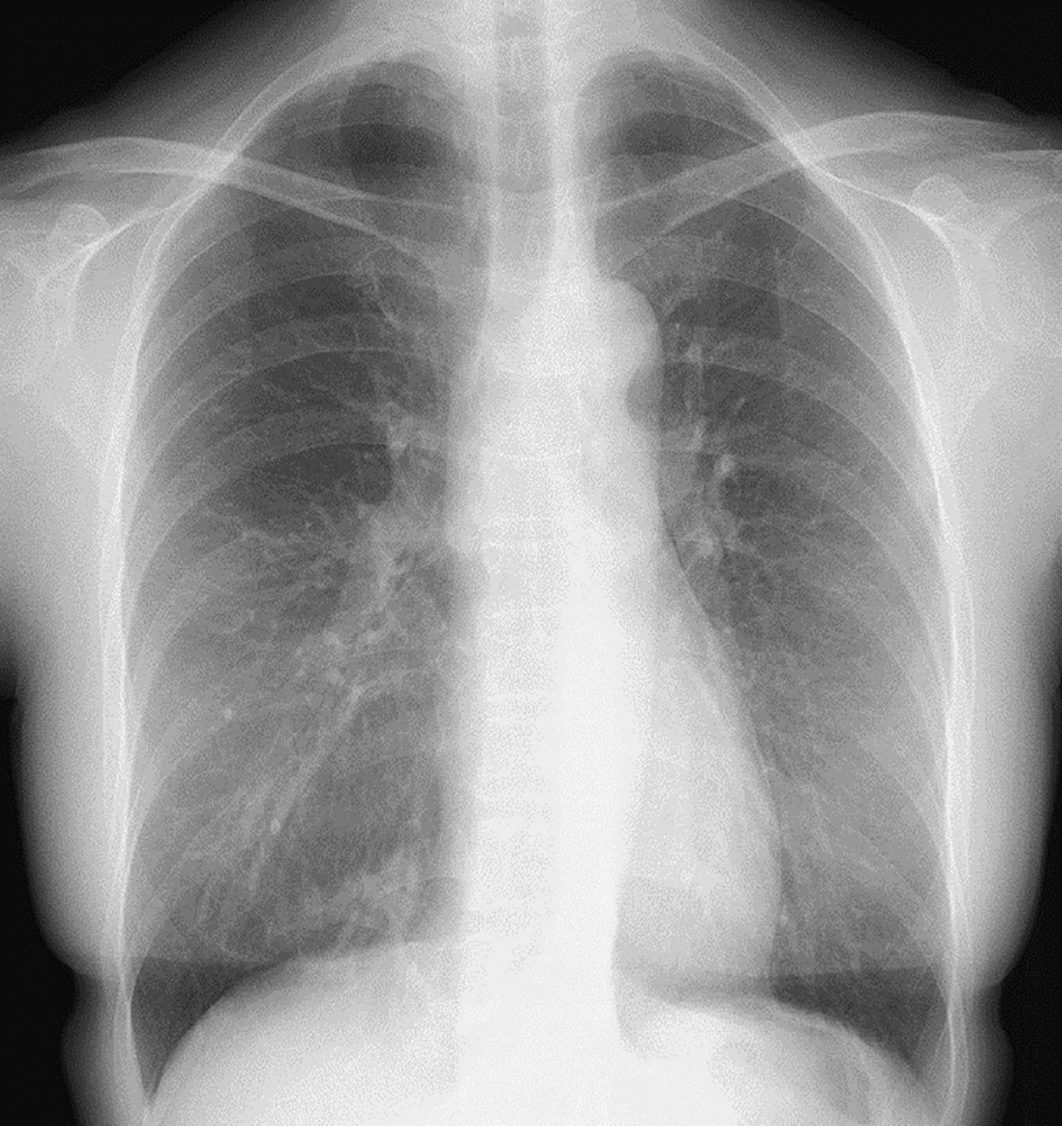
Chest x‐ray demonstrating multiple pulmonary nodules within the right lower lobe.

**FIGURE 2:**
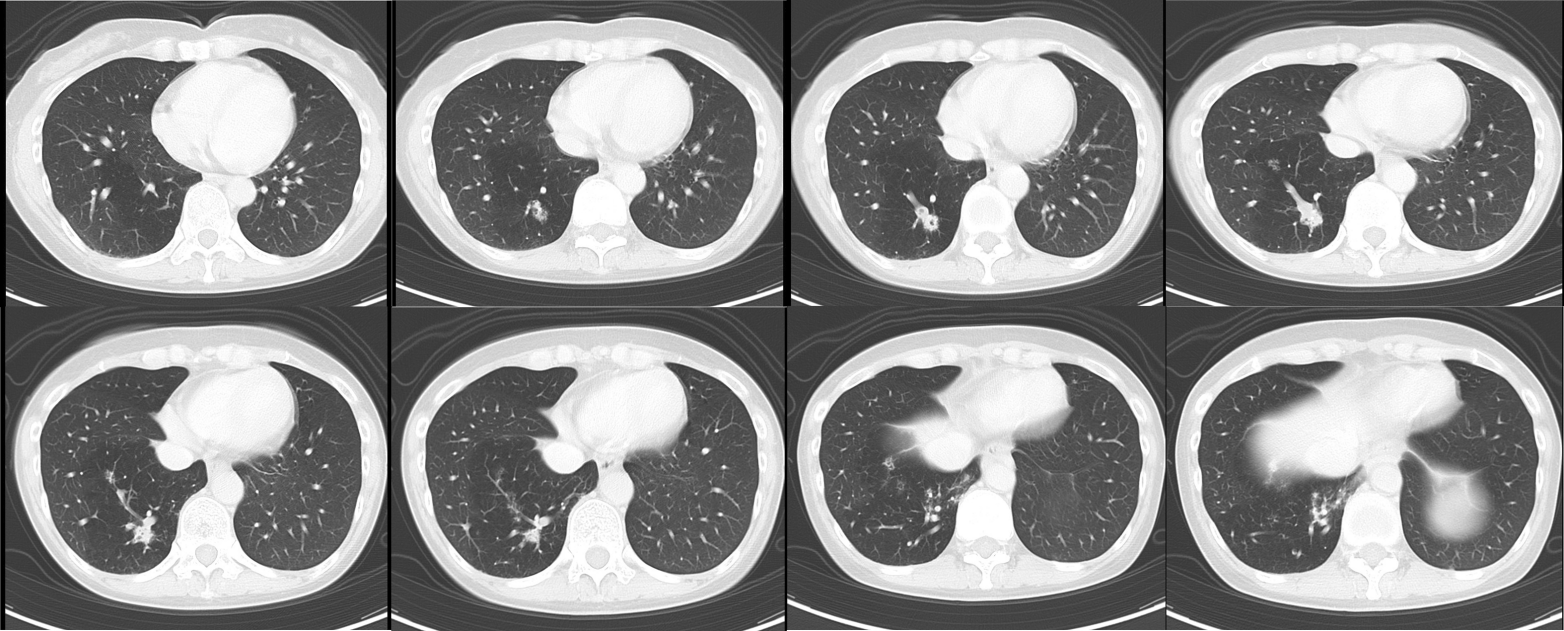
CE‐CT demonstrating multiple, nodular, pulmonary lesions with calcification in the posterior basal segment of the right lower pulmonary lobe with surrounding, focal emphysema.

**FIGURE 3:**
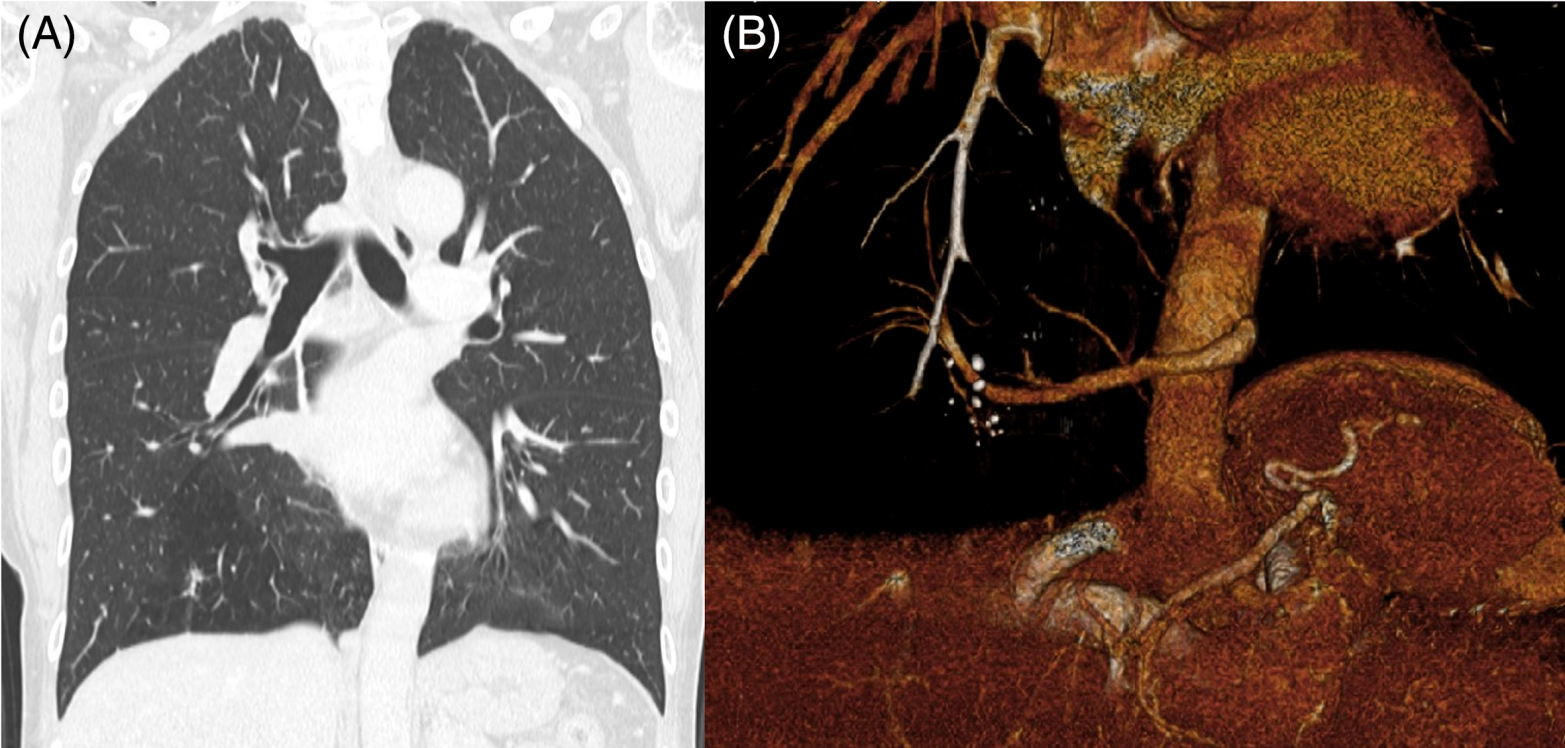
Coronal view showing an absence of communication with the tracheobronchial tree (A). 3D reconstruction demonstrating that the sequestrated lung segment had a systemic, arterial blood supply originating in the descending thoracic aorta and a venous return into the right pulmonary vein (B).

**FIGURE 4:**
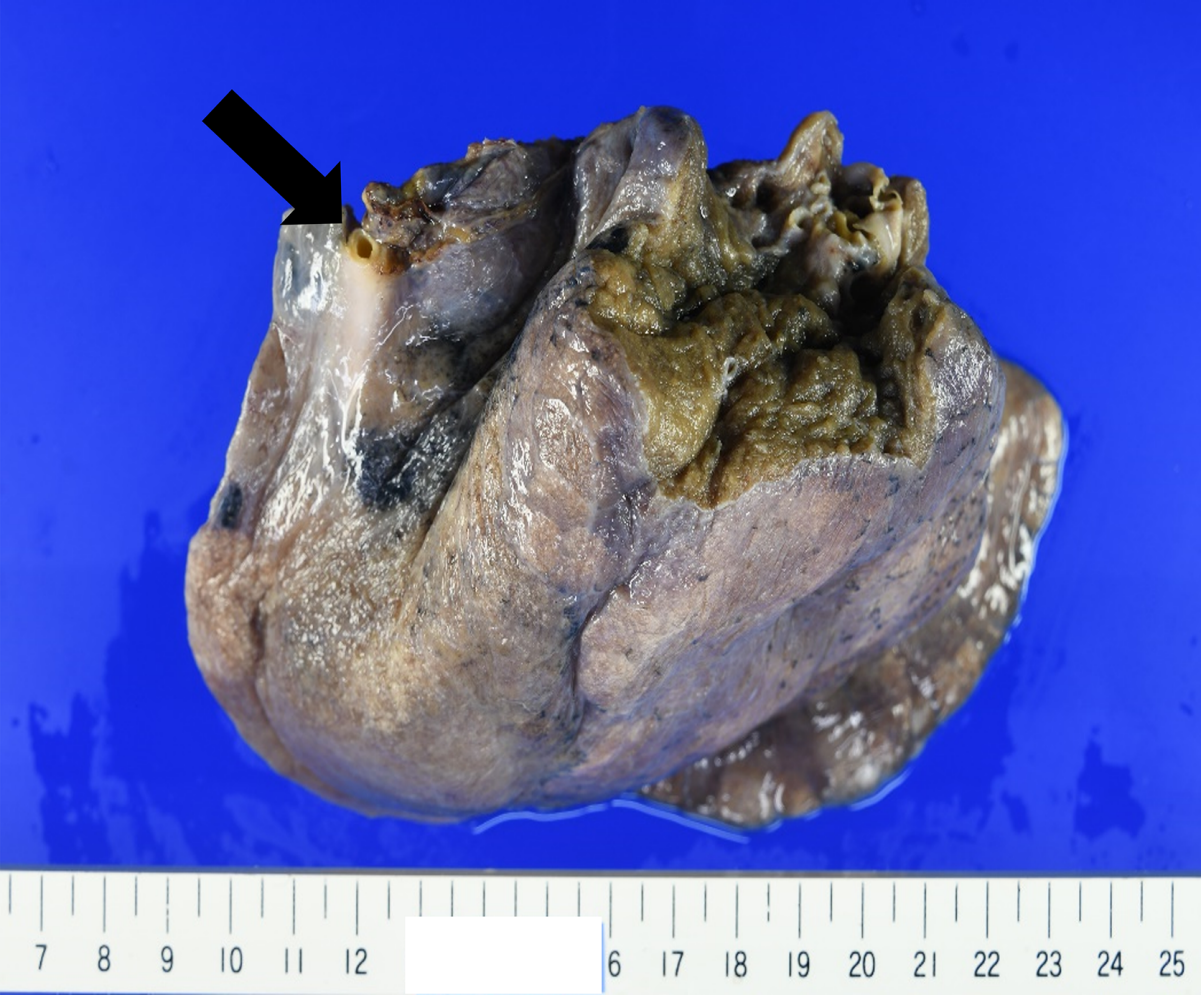
Pathological specimen. The ligated anomalous artery is indicated with a black arrow.

A 55‐year‐old, female patient presented with right back pain of 1 week's duration. She denied cough, hemoptysis, and fever and had an unremarkable past medical history. She had never smoked, and her physical examination and laboratory test findings were also unremarkable. Chest x‐ray revealed multiple, pulmonary nodules within the right lower lobe (Figure 1). Further investigation with CE‐CT demonstrated multiple, nodular, pulmonary lesions with calcification in the posterior basal segment of the right lower pulmonary lobe with surrounding focal emphysema (Figure 2). The pulmonary lesions suggested recurrent infections. There was no communication with the tracheobronchial tree on the coronal view, and 3D reconstruction demonstrated a systemic, arterial blood supply to the region originating from the descending thoracic aorta and venous return into the right pulmonary vein (Figure 3). The diameter of the anomalous artery was approximately 1 cm. The abnormal lung parenchyma had no pleura. These findings suggested ILS. A lung function test demonstrated normal spirometry values, and an echocardiogram found no pulmonary hypertension. The decision was made to perform a basal segmentectomy of the right lower lobe to remove the affected lung segment to prevent infection recurrence. Intraoperatively, an anomalous feeding artery was found (Figure 4). The patient had no complications after the segmentectomy and was discharged on postoperative day 7. She was still asymptomatic when examined at postoperative week 12.

## DISCUSSION

The following, two important clinical points can be gleaned from the present case. First, ILS can present multiple, nodular, pulmonary lesions with focal emphysema. Second, CE‐CT with multiplanar and 3D reconstruction is useful in diagnosing this condition.

Regarding the first point, the CT findings of ILS vary due to inflammation and infection. ILS typically presents a consolidation, solid mass, cystic lesion or cavity lesion with or without an air‐fluid level within a lower lobe.^6^ A previous, retrospective study found that the main CT finding in patients with ILS was a solid mass, with the second most common finding being cystic lesions, followed by cavitary lesions in third, pneumonic lesions in fourth; multiple, nodular, pulmonary lesions with focal emphysema rarely occurs.^7^, ^8^ The clinical symptoms of ILS also vary, and some patients present atypical symptoms, such as recurrent cough, hemoptysis, fever, chest pain or back pain. Many clinical symptoms and CT findings overlap with those of other respiratory diseases, such as recurrent infections, lung abscess, lung cancer, and lung cysts. Therefore, ILS may be incorrectly diagnosed or overlooked even with modern, radiological, imaging technology.

In the present case, the multiple, pulmonary nodules with calcification suggested recurrent infections. The focal emphysema surrounding the lesions resulted from collateral air ventilation through the pores of Kohn and air trapping owing to the lack of communication with the tracheobronchial tree. These CT findings should raise the index of suspicion of ILS.

Second, CE‐CT with multiplanar and 3D reconstruction, which is instrumental in identifying a systemic, arterial blood supply, is useful for accurately diagnosing this condition. Normally, angiography is the chief method used for this purpose, but recently, CE‐CT with multiplanar and 3D reconstruction has begun to be used as a more accurate, non‐invasive method of achieving this aim.^5^ A previous, retrospective study involving CE‐CT with 3D reconstruction in patients with a pathological diagnosis of PS found that this modality was able to identify the systemic, arterial blood supply highly accurately (100%); the multiplanar view enables accurate confirmation of the absence of communication with the tracheobronchial tree.^8^ In patients with several respiratory symptoms, CT is often performed to confirm the diagnosis. If recurrent infections within a lower lobe and the accompanying, radiological findings suggestive of ILS are confirmed on CT, multiplanar and 3D reconstruction should be performed additionally.

In conclusion, ILS can present pulmonary, nodular, pulmonary lesions with focal emphysema, and CE‐CT with multiplanar and 3D reconstruction is highly useful in diagnosing this condition. It should be borne in mind that ILS can present multiple, nodular, pulmonary lesions with focal emphysema rather than a mass or cystic lesions. Some cases may be overlooked owing the variability of the presentation. Further reports are needed to confirm whether some cases of ILS are more susceptible to misdiagnosis than others and whether routine CE‐CT with multiplanar and 3D reconstruction may contribute to reducing the rate of misdiagnosis.

## AUTHOR CONTRIBUTIONS

Tadatsugu Yoshinaga wrote the first draft, and Makiko Yomota, Kazutoshi Toriyama, Hirokazu Iso, Kie Mirokuji, Syoko Kawai, Kosuke Narita, Mikito Suzuki, Hirotoshi Horio, and Yukio Hosomi revised the manuscript for important intellectual content. All the authors approved the final version.

## CONFLICT OF INTEREST STATEMENT

None declared.

## ETHICS STATEMENT

The authors declare that appropriate written informed consent was obtained for the publication of this manuscript and accompanying images.

## Data Availability

All relevant data are within the manuscript.
